# The mismatch-repair proteins MSH2 and MSH6 interact with the imprinting control regions through the ZFP57-KAP1 complex

**DOI:** 10.1186/s13072-022-00462-7

**Published:** 2022-08-02

**Authors:** Basilia Acurzio, Francesco Cecere, Carlo Giaccari, Ankit Verma, Rosita Russo, Mariangela Valletta, Bruno Hay Mele, Claudia Angelini, Angela Chambery, Andrea Riccio

**Affiliations:** 1 Department of Environmental Biological and Pharmaceutical Sciences and Technologies (DiSTABiF), Università Degli Studi Della Campania “Luigi Vanvitelli”, 81100 Caserta, Italy; 2 Institute of Genetics and Biophysics (IGB) “Adriano Buzzati-Traverso”, Consiglio Nazionale Delle Ricerche (CNR), 80131 Naples, Italy; 3 Department of Biology, Università Degli Studi Di Napoli “Federico II”, 80126 Naples, Italy; 4 Istituto Per Le Applicazioni del Calcolo “Mauro Picone” (IAC), CNR, 80131 Naples, Italy

**Keywords:** LC–MS/MS, Chip-seq, Allele-specific analysis, CpG islands, Methyl CpG, Genomic imprinting, Mismatch-repair, Transcription factor binding, Cytosine deamination

## Abstract

**Background:**

Imprinting Control Regions (ICRs) are CpG-rich sequences acquiring differential methylation in the female and male germline and maintaining it in a parental origin-specific manner in somatic cells. Despite their expected high mutation rate due to spontaneous deamination of methylated cytosines, ICRs show conservation of CpG-richness and CpG-containing transcription factor binding sites in mammalian species. However, little is known about the mechanisms contributing to the maintenance of a high density of methyl CpGs at these loci.

**Results:**

To gain functional insights into the mechanisms for maintaining CpG methylation, we sought to identify the proteins binding the methylated allele of the ICRs by determining the interactors of ZFP57 that recognizes a methylated hexanucleotide motif of these DNA regions in mouse ESCs. By using a tagged approach coupled to LC–MS/MS analysis, we identified several proteins, including factors involved in mRNA processing/splicing, chromosome organization, transcription and DNA repair processes. The presence of the post-replicative mismatch-repair (MMR) complex components MSH2 and MSH6 among the identified ZFP57 interactors prompted us to investigate their DNA binding profile by chromatin immunoprecipitation and sequencing. We demonstrated that MSH2 was enriched at gene promoters overlapping unmethylated CpG islands and at repeats. We also found that both MSH2 and MSH6 interacted with the methylated allele of the ICRs, where their binding to DNA was mediated by the ZFP57/KAP1 complex.

**Conclusions:**

Our findings show that the MMR complex is concentrated on gene promoters and repeats in mouse ESCs, suggesting that maintaining the integrity of these regions is a primary function of highly proliferating cells. Furthermore, the demonstration that MSH2/MSH6 are recruited to the methylated allele of the ICRs through interaction with ZFP57/KAP1 suggests a role of the MMR complex in the maintenance of the integrity of these regulatory regions and evolution of genomic imprinting in mammalian species.

**Supplementary Information:**

The online version contains supplementary material available at 10.1186/s13072-022-00462-7.

## Background

Genomic imprinting is a gene regulatory mechanism of mammals based on differential acquisition of epigenetic marks during female and male gametogenesis [1]. These marks oppositely influence the expression of the maternally inherited and paternally inherited alleles of nearby genes at later stages of development. The best characterized form of imprinted gene modification is CpG methylation, and most imprinting control regions (ICRs) identified so far overlap CpG-rich sequences (germline-derived Differentially Methylated Regions), whose methylation is differentially established in female and male germ cells and faithfully maintained in somatic cells throughout development.

Maintenance of differential methylation of the ICRs is particularly critical during pre-implantation development, a stage in which epigenetic reprogramming occurs. Several human diseases (overall known as imprinting disorders) arise from loss or gain of methylation at specific imprinted loci at this stage [2]. A multi-protein complex including the zinc-finger protein ZFP57 and its cofactor KAP1 specifically recognizes the methylated allele of the ICRs and is required for maintaining their DNA methylation in mouse embryos and embryonic stem cells [3–6]. ZFP57/KAP1 binding is also necessary for maintaining different histone marks on the maternal and paternal alleles of the ICRs and allele-specific expression of the imprinted genes [7–10]. In humans, biallelic loss of function mutations of *ZFP57* lead to Transient Neonatal Diabetes type 1 and multi-locus imprinting disturbances [11]. While ZFP57 recognizes multiple methylated CpG-containing motifs on one allele of the ICRs, the other and non-methylated allele of these regulatory regions is bound by several other transcription factors required for preventing de novo methylation and promoting transcription of the imprinted genes [2].

The mutation rate of CpGs in mammalian genomes is generally elevated because these dinucleotides are often methylated, and methylated cytosine is unstable as it undergoes deamination to thymine, which if uncorrected yields a C to T transition [12]. This mechanism is mostly responsible for the lower frequency of the CpG dinucleotide in mammalian genomes. An exception is represented by the CpG islands that overlap gene promoters and are generally non-methylated in the germline, thus preserving their CpG-richness. Although the ICRs are methylated on one allele in the germline, they maintain their characteristics of 2–4 kb long CpG-rich regions and show conservation of CpG-containing transcription factor binding sites in mammals [13, 14].

The mismatch-repair (MMR) complex recognizes misincorporated bases in double-stranded DNA and is required to repair G:T mis-matches resulting from deamination of 5-methylcytosine (5mC) [15]. It is worth noting that mutations arising from unrepaired 5mC deamination events are prevalent in MMR-deficient cancers, particularly those deficient in the MutSα heterodimer that is composed by the MSH2-MSH6 heterodimer [15].

To gain further insights into the mechanisms of maintenance of methyl CpGs in the ICRs, we sought to identify the proteins interacting with the methylated allele of the ICRs, by determining the ZFP57 interactors in mouse ESCs. We were able to identify several factors involved in mRNA processing/splicing, chromosome organization, transcription and DNA repair processes. We focused on the components of the mismatch-repair complex MSH2 and MSH6 and demonstrated their interactions with the ICRs by investigating their DNA binding profile by chromatin immunoprecipitation and sequencing. Although MSH2 was found preferentially enriched at non-methylated CpG islands, both MSH2 and MSH6 also showed interaction with the methylated allele of the ICRs through the ZFP57/KAP1 complex.

## Results

### Identification of ZFP57-interacting proteins by LC–MS/MS analysis

**Fig. 1:**
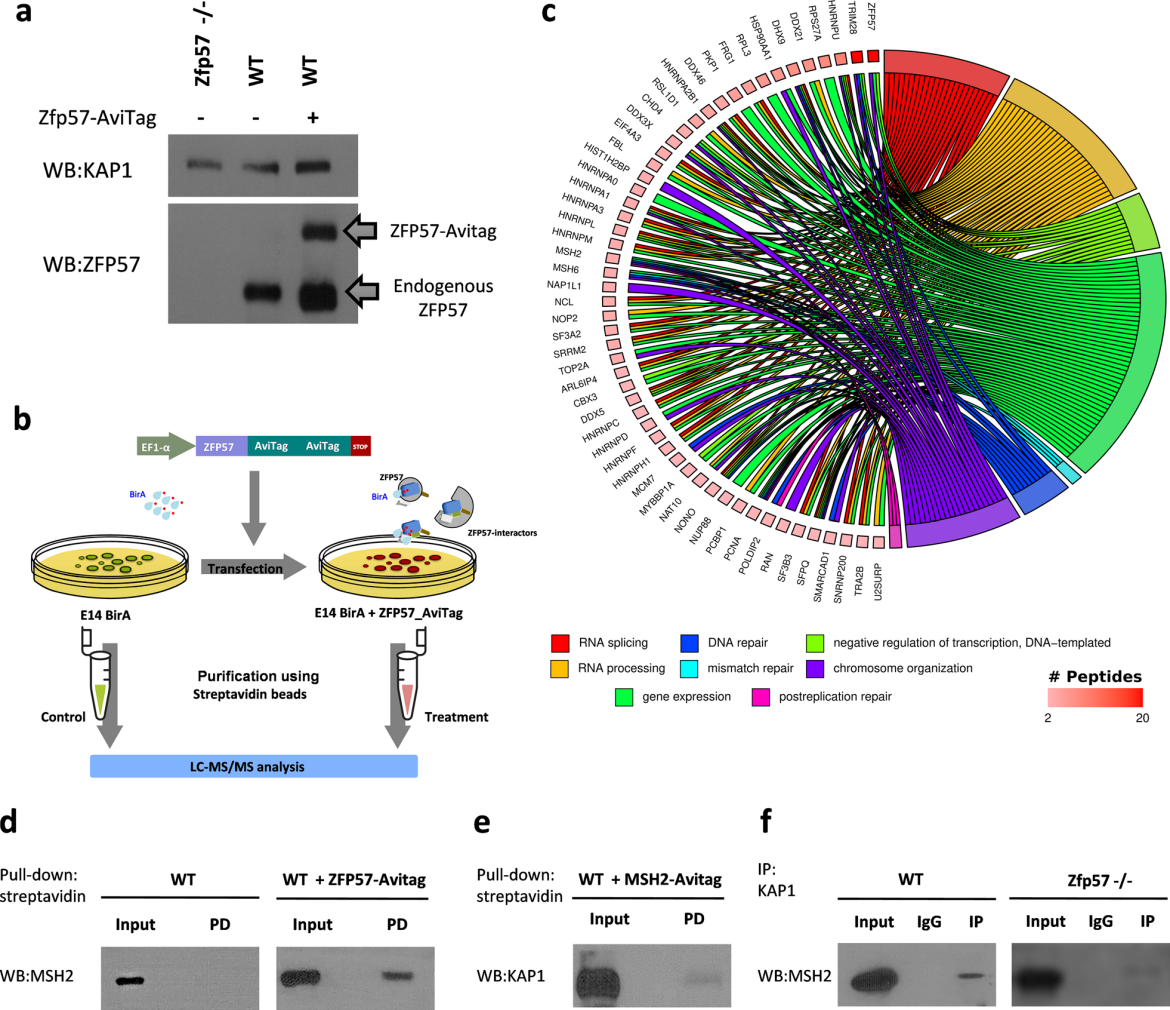
High-confidence interaction partners of ZFP57 in mouse ESCs. **a** Western blot analysis of ZFP57 and KAP1 in wild-type (WT), *Zfp57*-AviTag-transfected and *Zfp57*-/- ESCs. Note that AviTag-ZFP57 migrates as a 60 kDa band while the endogenous ZFP57 migrates as a 50 kDa-band. **b** Experimental workflow of the tagged protein-mass spectrometry approach used for identification of the ZFP57 interactors. The *Zfp57* cDNA was cloned into the expression vector pEF6-Avitag-GGGx2-Avitag. *Zfp57*-AviTag was transfected in stably BirA-expressing mouse ESCs and biotin-labelled pulled proteins were pulled down using streptavidin. Precipitated protein complexes were digested with trypsin for LC–MS/MS analysis. Proteins in common between *Zfp57*-AviTag-transfected and Mock-transfected BirA-expressing E14 ESCs were excluded from further analyses. Images of petri dishes and eppendorf tubes are taken from https://doi.org/10.7875/togopic.2020.104 and https://doi.org/10.7875/togopic.2022.115, respectively. BirA is depicted in light blue, biotin in red, ZFP57 in dark blue and ZFP57-interactors in grey, green, and olive. *Zfp57*-AviTag-transfected and mock-transfected BirA-expressing E14 ESCs are depicted in red and green, respectively. **c** Chord diagram showing enriched GO clusters of ZFP57 interactors. The proteins identified by LC–MS/MS analysis ordered according to their relative enrichment are shown on the left, and the enriched GO clusters are indicated with different colours on the right. The number of peptides by which proteins were recognized by LC–MS/MS analysis is displayed in gradient red (2–20 peptides). **d** Western blot of proteins pulled down with streptavidin in WT and *Zfp57*-AviTag-transfected ESCs and revealed with anti-MSH2 antibody. **e** Western blot of proteins pulled down with streptavidin in *Msh2*-AviTag-transfected ESCs and revealed with anti-KAP1 antibody. **f** Western blot of proteins immunoprecipitated with anti-KAP1 antibody in WT and Zfp57-/- ESCs and revealed with anti-MSH2 antibody. Input corresponds to 1% of the cell lysate used for immunoprecipitation

Because of the poor specificity and high background detected with commercial ZFP57-specific antibodies, we used a tagged approach to identify the proteins interacting with ZFP57. We chose the Avi-Tag that can be labeled with biotin in cells expressing the bacterial biotin ligase BirA and subsequently pulled down with streptavidin [16]. This method was applied to the mouse embryonic stem cells (ESCs) E14, in which the endogenous *Zfp57* gene is highly expressed and functional [4]. Thus, BirA-expressing E14 ESCs were transfected with the tagged *Zfp57* gene (*Zfp57-*AviTag) (Fig. 1a) and the proteins interacting with the biotinylated ZFP57-AviTag were pulled down in cell lysates with streptavidin-coated beads, purified and analyzed by mass spectrometry. To exclude the proteins that could be aspecifically biotinylated by BirA, we also analyzed mock-transfected BirA-expressing E14 ESCs. Only the proteins exclusively detected in the *Zfp57-*AviTag-transfected cells (Additional file 1: Table S1a) were considered for further analyses. A schematic outline of this procedure is shown in Fig. 1b. LC–MS/MS analysis allowed us to identify 60 high-confidence ZFP57-interacting proteins (Additional file 1: Table S1a). This list includes KAP1 and HP1γ, which were already reported as ZFP57 interactors [4, 17], as well as several novel proteins. A gene ontology (GO) analysis allowed classifying the ZFP57 interactors into several functional categories, including mRNA processing/splicing, chromosome organization, transcription and DNA repair processes (Fig. 1c). By using the STRING database [18], we reconstructed a network model of the ZFP57-interacting proteins (Additional file 2: Fig. S1a). This approach allowed us to identify several protein families or complexes potentially interacting with ZFP57, including histone H1 variants, Heterogeneous Nuclear Ribonucleoproteins and the post-replicative DNA Mismatch-Repair (MMR) complex including MSH2, MSH6 and PCNA.

We were particularly interested in the interaction of the MSH2/MSH6 heterodimer of the MMR complex with ZFP57, because of its potential role in CpG conservation at the ICRs. We then validated the interaction of MSH2 with the ZFP57-AviTag by co-precipitation and Western blotting (Fig. 1d). Similarly, we demonstrated that KAP1 interacts with the Avi-tagged MSH2 (Fig. 1e), that the endogenous MSH2 and KAP1 proteins interact in the wildtype E14 ESCs and that this interaction is maintained in the *Zfp57*-/- ESCs (Fig. 1f). Overall, these results confirm the interaction of the DNA mismatch repair complex with ZFP57 and indicate that this is probably mediated by KAP1.

### DNA-binding profile of MSH2

**Fig. 2:**
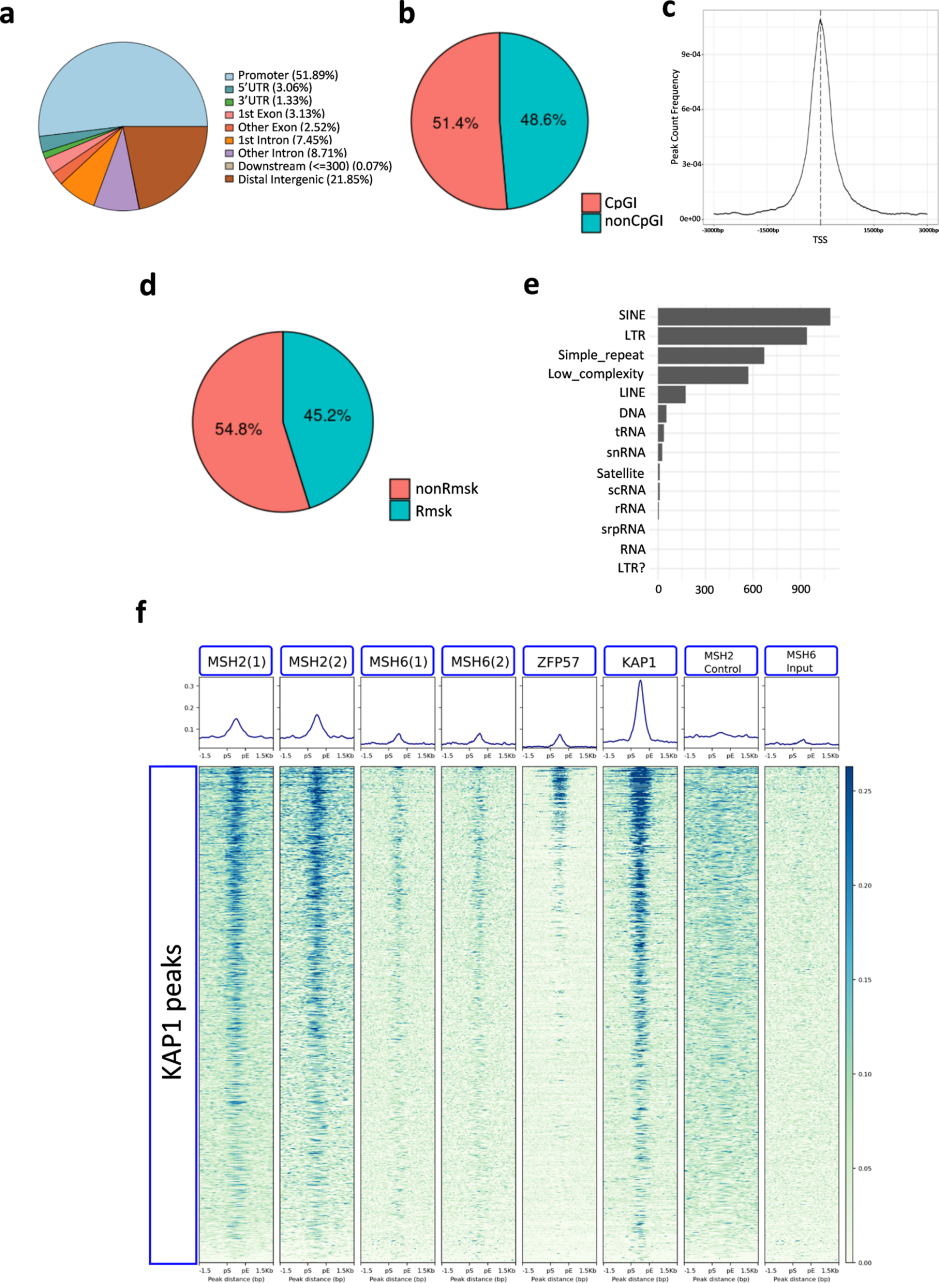
Genome-wide binding profiles of MSH2 and MSH6 in mouse ESCs as revealed by Bio-ChIP-seq and ChIP-seq. **a** Pie chart showing the distribution of genomic elements within the DNA regions covered by MSH2 Bio-ChIP-seq peaks. MSH2 peaks were defined as DNA regions that were enriched in the *Msh2*-AviTag cells compared to mock-transfected BirA-expressing ESCs. For feature annotation, we used the plotAnnoPie function with annoDb = "org.Mm.eg.db" parameter". **b** Pie chart representing the percentage of MSH2 Bio-ChIP-seq peaks overlapping CpG islands (CpGI) and genomic regions not including CpGI (non CpGI). **c** Average Profile of MSH2 peaks overlapping Transcription Start Sites (TSS). The plot shows the peak frequency between −3000 bp and + 3000 bp from TSS. **d** Pie chart representing the percentile distribution of MSH2 peaks overlapping or not overlapping mask repetitive elements. **e** Bar plot showing the absolute frequency of repetitive elements covered by MSH2 peaks. **f** Heatmaps showing the read enrichment of MSH2 (2 replicates), MSH6 (2 replicates), ZFP57 and KAP1 in the genomic regions overlapping (± 1.5 kbp) the KAP1 ChIP-seq peaks. As controls, we added the Bio-ChIP-seq reads of untransfected E14 ESCs (MSH2 control) and input DNA reads of the MSH6 ChIP (MSH6 input). KAP1 peaks are sorted in descending order based on the mean read enrichment value per region

After validating the interaction between MSH2 and the ZFP57-KAP1 complex, we determined the DNA binding profile of MSH2 in mouse ESCs. We used the approach based on proteins fused to AviTag and pulldown with streptavidin. This method has been demonstrated to be efficient for chromatin immunoprecipitation and sequencing (Bio-ChIP-seq), particularly when the available antibodies raised against the endogenous protein produce high background [19]. Application of the Bio-ChIP-seq protocol to determine MSH2 binding in the E14 ESCs revealed 4444 shared peaks between two replicates (Additional file 1: Tables S2a and S2b). The genomic regions mostly enriched by MSH2 corresponded to gene promoters (52%) and CpG islands (CpGI, 51,4%) and most of the peaks overlapped transcription start sites (TSS, Fig. 2a–c and Additional file 2: Fig. S2), including the promoters of several cell growth-controlling genes, such as *Jun*, *Jund*, *Fos* and *Nras* (Additional file 2: Fig. S3). In addition, about 45% of the MSH2 peaks overlapped repetitive sequences, of which the most abundant categories were SINEs and LTRs (Fig. 2d, e).

To better characterize the relationship between the MMR proteins and the ZFP57/KAP1 complex, we analysed the distribution of MSH2 ChIP-seq reads along 1343 KAP1 ChIP-seq peaks [7] in the E14 ESCs. This analysis revealed co-localization of many MSH2 peaks with ZFP57 and KAP1 peaks (Fig. 2f). The overlap between MSH2 and KAP1 primarily involved intergenic and noncoding regions and generally no promoter CpGI (Additional file 2: Figs. S3 and S4). However, enrichment of both MSH2 and KAP1 was particularly intense at the ICRs and non-ICR ZFP57 binding sites (Additional file 2: Figs. S5 and S6). In addition, MSH2 bound many KAP1 binding sites that are not targeted by ZFP57 supporting the hypothesis that MSH2 interacts with KAP1 independently of ZFP57 (Additional file 2: Fig. S5). We also performed a ChIP-seq experiment for MSH6 in the E14 ESCs by using the antibodies raised against the native protein. The cells were treated with oxygen peroxide to increase the recruitment of MSH6 to DNA [20]. Although very few peaks could be discriminated with this antibody, the alignment of the MSH6 ChIP-seq reads along the KAP1 peaks demonstrated MSH6 enrichment on the KAP1 binding sites, confirming the results obtained with MSH2 (Fig. 2f and Additional file 2: Figs. S4 and S5).

These results demonstrate that MSH2 is mostly enriched on promoter CpGIs and repeats. Furthermore, we showed that the sequences bound by both MSH2 and MSH6 overlap those bound by ZFP57 and KAP1 in mouse ESCs.

### Interaction of MSH2 and MSH6 with the ICRs

**Fig. 3:**
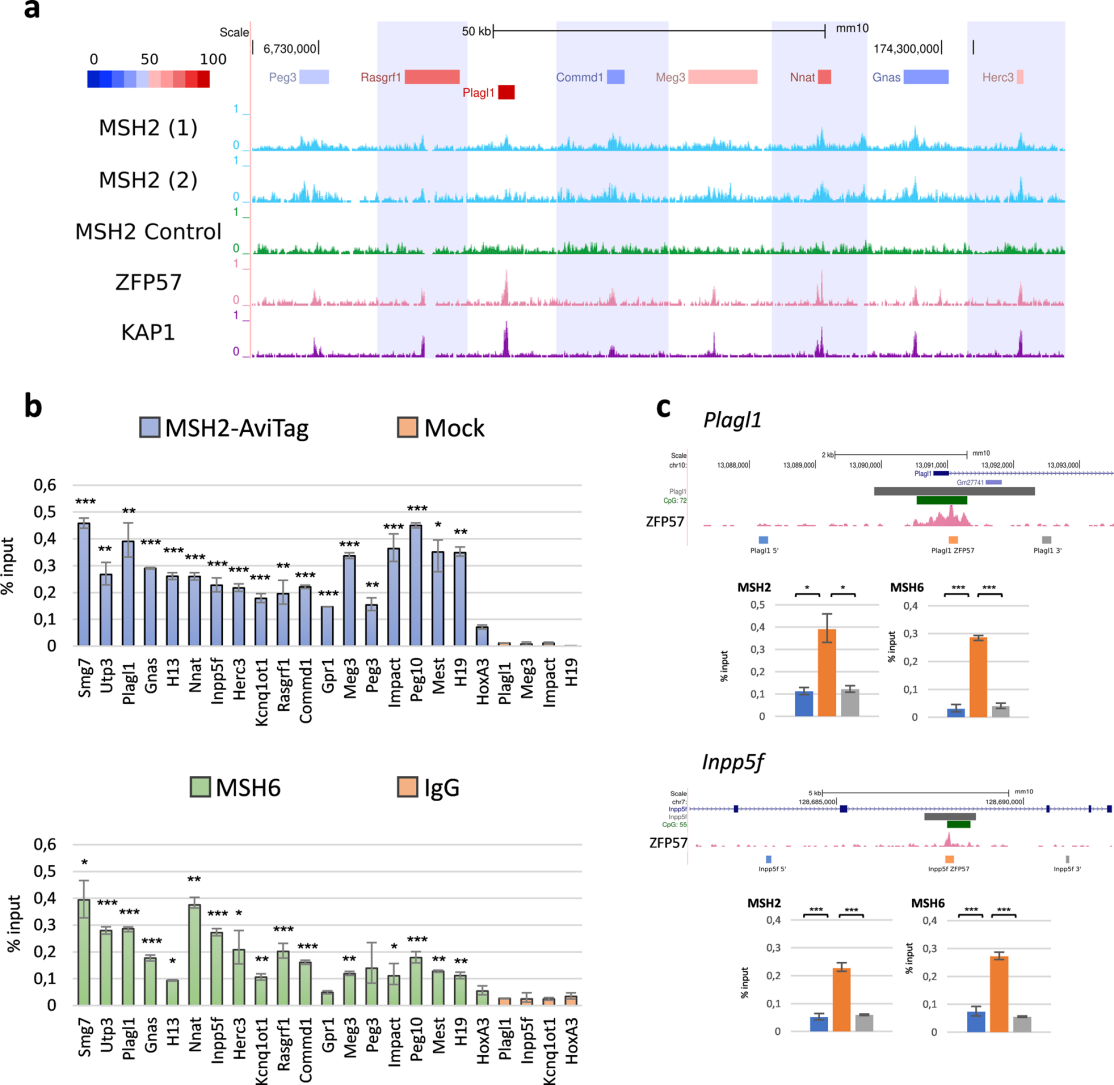
Characterization of MSH2 and MSH6 binding at the ICRs. **a** Screen shots from the UCSC Genome Browser showing the Bio-ChIP-seq peaks detected for Biotin-tagged MSH2 in BirA-expressing E14 ESCs along 8 ICRs compared with previously determined ZFP57 and KAP1 binding regions. The ICRs are shown as horizontal boxes coloured according to the mean DNA methylation level determined by RRBS. Enrichment of Bio-ChIP-seq reads in *Msh2*-AviTag-transfected ESCs (2 replicates) and mock-transfected BirA-expressing ESCs (MSH2 control) are shown in light blue and green, respectively; enrichment of ZFP57 and KAP1 ChIP-seq reads are in pink and purple, respectively. **b** Bar-plots showing the relative enrichment of Biotin-tagged MSH2 (blue) and MSH6 (green) at ICRs as determined by Bio-ChIP and ChIP, respectively, in WT E14 ESCs. The *Smg7* and *Utp3* promoter CpGI were used as positive controls, while a region of the *Hoxa3* gene that is not overlapping a CpGI and showed lower enrichment of MSH2 by Bio-ChIP-seq was used as negative control. ChIP values are expressed as % input. Error bars represent the SD of three biological duplicates. Significant enrichment of MSH2-AviTag was demonstrated by Bio-ChIP at ICRs in *Msh2*-AviTag-transfected with respect to mock-transfected BirA-expressing ESCs (Mock), and significant enrichment of MSH6 was demonstrated at ICRs with anti-MSH6 antibodies with respect to IgG. **c** Bar-plots showing the relative enrichment of MSH2 and MSH6 at the *Plagl1* and *Inpp5f* ICRs and flanking regions determined as in b. Screen-shots from the UCSC Genome Browser showing features of the analysed regions are reported above the bar-plots. The ICRs are indicated in dark grey, CpGI in green, ZFP57 peaks in pink. Orange bars represent the ICRs overlapping the ChIP-seq peaks of ZFP57. Blue, orange and light grey bars indicate the location of the primers used for Q-PCR. ChIP values are expressed as % input. Error bars represent the SD of two biological replicates each averaged over two technical replicates. *p < 0.05, **p < 0.01, ***p < 0.001

We then focused on the imprinted loci and found that MSH2 binding revealed by Bio-ChIP-seq was enriched on 10 ICRs in at least one replicate and the MSH2 peaks coincided with the ZFP57 and KAP1 peaks in these regions (Fig. 3a and Additional file 1: Table S2a, b). Because of the relatively high background observed with the MSH2 Bio-ChIP-seq, we validated the binding of MSH2 and tested that of MSH6 to the ICRs by quantitative PCR after precipitation of MSH2 by Bio-ChIP and MSH6 by conventional ChIP, respectively. The results demonstrated higher enrichment of MSH2 and MSH6 on 16 and 14 ICRs, respectively, when compared with the *Hoxa3* gene that does not overlap with CpGI (Fig. 3b) and ICR-adjacent sequences (Fig. 3c).

### MSH2 and MSH6 bind the methylated ICR allele in a Zfp57-dependent manner

**Fig. 4:**
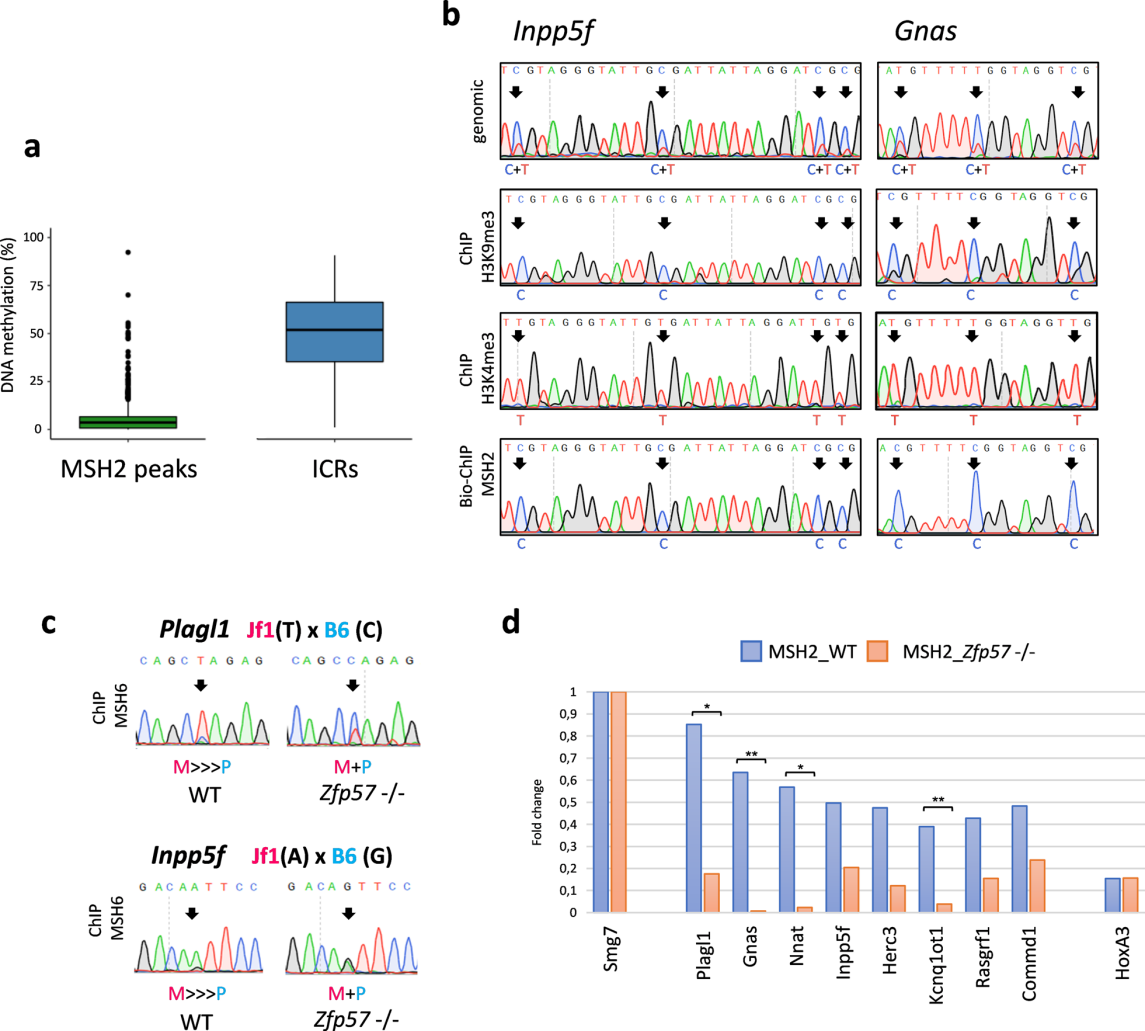
Parental origin-specific binding of MSH2 and MSH6 to the ICRs. **a** Box plot showing the methylation level of non-repetitive MSH2-bound regions (left) and MSH2-bound ICRs (right). **b** Electropherograms showing the DNA methylation level of the *Inpp5f* and *Gnas* ICRs in genomic DNA, ChIP DNA obtained with anti-H3K9me3, anti-H3K4me3 antibodies and Bio-ChIP DNA pulled down with streptavidin in *Msh2*-AviTag-transfected ESCs, and revealed by bisulfite sequencing. Methylation levels were determined from the ratio between methylated (C) and unmethylated (T) cytosines. **c** Electropherograms showing the allele-specific binding of MSH6 in hybrid WT and *Zfp57*-/- ESCs at the *Plagl1* and *Inpp5f* ICRs. The SNVs associated with the maternal JF1 or paternal Black/6 (B6) alleles are shown at the top. The relative enrichment of the maternal over the paternal allele is reported below the electropherogram. Black arrows indicate the SNVs. **d** Bar plot showing relative enrichment of Biotin-tagged MSH2 protein to ICRs in WT and *Zfp57*-/- ESCs. ChIP values were expressed as % input and normalized to the *Smg7* region that is used as a positive control

To investigate the methylation status of the sequences bound by MSH2 and MSH6, we determined the whole-genome methylation profile of the E14 ESCs by RRBS. We found that most of the non-repetitive MSH2-bound regions that could be analyzed with this method showed low methylation levels (Fig. 4a). The only exceptions were the ICRs that displayed an average methylation level of 50%, consistent with their imprinting status, and a few other non-imprinted ZFP57-bound loci in most of which RRBS analysis demonstrated higher methylation levels (Figs. 3a and 4a and Additional file 2: Fig. S6).

Because the ICRs have one allele methylated and the other allele non-methylated, we asked if there was any bias in the binding of MSH2 and MSH6 to these genomic regions. To discriminate between methylated and unmethylated cytosines, we treated the MSH2-bound DNA precipitated with the Bio-ChIP protocol in the E14 ESCs with sodium bisulfite and analyzed it by Sanger sequencing. We observed that the methylated allele of the *Inpp5f* and *Gnas* ICRs was preferentially enriched in the MSH2-bound DNA (Fig. 4b). As a control, we tested the methylation status of the *Inpp5f* and *Gnas* ICR sequences that were recovered after ChIP with anti-H3K9me3 and anti-H3K4me3 antibodies, and found that H3K9me3 and H3K4me3 were preferentially enriched on the methylated and non-methylated allele, respectively. This observation is consistent with previous results [7]. To determine if also MSH6 interacts with the DMRs in allele-specific manner, we performed a ChIP experiment in an ESC line (JB1) derived from an intra-specific mouse hybrid (JF1x C57-Black/6). In this ChIP, the maternal and paternal alleles of the ICRs can be discriminated through JF1- and C57-Black/6-specific SNVs [10]. Using this approach, we showed that MSH6 was preferentially enriched on the maternal JF1 allele of the *Plagl1* and *Inpp5f* ICRs in the JB1 ESCs (Fig. 4c). Because the *Plagl1* and *Inpp5f* ICRs are methylated on the maternal allele [10], these results confirm those obtained with MSH2.

Since the binding sites of MSH2 and MSH6 overlap those of ZFP57, we asked if the enrichment of these proteins at the ICRs depended on this zinc-finger protein. To address this issue, we first investigated MSH2 binding to the ICRs in wildtype and *Zfp57*-/- E14 ESCs by Bio-ChIP. The results demonstrated that MSH2 enrichment was significantly reduced on eight ICRs, upon losing the *Zfp57* gene (Fig. 4d). The role of ZFP57 on MSH6 binding was investigated in the JB1 ESCs by conventional ChIP. Sanger sequencing of the DNA immunoprecipitated with anti-MSH6 antibodies demonstrated that the allelic bias in MSH6 binding to the *Plagl1* and *Inpp5f* ICRs observed in the wildtype JB1 ESCs was lost in the *Zfp57* -/- JB1 ESCs (Fig. 4c).

In summary, these results demonstrate the preferential interaction of MSH2 and MSH6 with the methylated allele of the ICRs and indicate that DNA binding is mediated by ZFP57.

## Discussion

The ICRs are DNA sequences with unique properties [1, 2]. Maintenance of parental origin-dependent methylation and allele-specific expression of the imprinted genes require the presence of multiple CpGs and the binding sites of transcription factors recognizing either methylated or non-methylated DNA on the two parental alleles of these regulatory sequences. We used the ZFP57 zinc-finger protein as bait to identify the proteins interacting with the methylated allele of the ICRs in mouse ESCs. By high-resolution mass spectrometry, members of the MMR complex were identified in the ZFP57 interactome. We explored the DNA binding profile of MSH2 and MSH6 and found that they were enriched at non-methylated CpGI, but they also bound the methylated allele of the ICRs. The analysis of *Zfp57*-deficient cells allows the conclusion that MSH2/MSH6 binding to the ICRs is mediated by ZFP57.

By employing an LC–MS/MS-based approach, we identified 60 potential ZFP57-interactors in mouse ESCs. Interestingly, all these proteins have previously been pulled down with anti-KAP1 antibodies in human ESCs and K562 cells, confirming that they are bona fide interactors of the ZFP57-KAP1 complex [21]. Furthermore, some of these interactions, such as those of the H3K9me3-interacting protein HP1γ and the chromatin remodelling factor CHD4 were characterized in previous studies [4, 22]. Among the novel ZFP57 interactors discovered in this study, histone H3.3 variants have been previously associated with the ICRs, while Histone H1 variants have been reported to recruit DNMTs to these regulatory regions in mouse ESCs [23]. Particularly interesting is also the finding of multiple Heterogeneous Nuclear Ribonucleoproteins including HNRNP-U, which may be involved in shaping the large-scale chromatin structures controlling parent-of-origin-dependent allelic gene expression at the imprinted loci [24, 25].

Our study identified three components (MSH2, MSH6 and PCNA) of the post-replicative MMR complex as ZFP57 interactors by LC–MS/MS analysis. The MMR pathway is essential for repairing 5mC deamination because the MSH2/MSH6 heterodimer recognizes G:T mismatches and recruits downstream proteins for correction [15, 26]. Indeed, defects of MMR genes increase the genome-wide mutation rate of methylated CpGs in cancer [15, 27]. Also, *Msh2/Msh6* knockout mice show higher cancer predisposition, microsatellite instability and mutator phenotype [28–31]. Thus, the interaction of MSH2/MSH6 with the methylated allele of the ICRs may reduce their mutation rate and preserve imprinting in rapidly dividing embryonic cells. A few methylated sequences were found among the MSH2 target sites. All of them correspond to ZFP57 binding sequences and show particularly high enrichment of MSH2 and KAP1, indicating specific MMR complex recruitment at the imprinted loci mediated by the ZFP57-KAP1 complex.

Upon oxidative damage, the MSH2-MSH6 heterodimer contributes to the recruitment of epigenetic silencing proteins, including DNMT1, SIRT1, and EZH2, to promoter CpGI in human cells [20]. This recruitment results in a transient reduction of transcription while the repair occurs. Our findings of MSH2 enrichment on TSS overlapping CpGI are consistent with these results. Interestingly, preferential MSH2/MSH6 binding to the promoters of cell growth-controlling genes suggests a safeguarding mechanism for rapidly proliferating cells. Furthermore, unlike the study performed by Ding and collaborators [20], our cells were untreated with oxygen peroxide before the MSH2 Bio-ChIPseq experiment, suggesting that MSH2/MSH6 also recognizes CpG-rich promoters in absence of oxidative damage. However, it should be considered that the increased MSH2 concentration after gene transfer may have favored its interaction with DNA in our cells. So, it is not possible to exclude that interaction of MSH2/MSH6 with CpG-rich promoters and ICRs occurs preferentially after DNA damage. Targeting of MSH2 to promoter CpGI appears generally not mediated by ZFP57/KAP1 and its underlying mechanisms need to be elucidated in future studies. Our results are not consistent with the interaction of MSH6 with gene bodies via H3K36me3 demonstrated in human cells, suggesting the occurrence of species- or cell type-specific differences [32].

The MSH2 ChIP-seq also revealed enrichment of MSH2 at repetitive elements, mostly SINEs, LTRs, low-complexity and simple sequence repeats. Some of these interactions may be mediated by KAP1, which is known to bind repetitive sequences and contribute to genomic stability [33, 34]. It is possible that these interactions contribute to the anti-mutator and anti-recombination functions of the MMR proteins [35].

In conclusion, by determining the genomic binding profile of MSH2/MSH6, our study provides novel insights into the function of the MMR complex and reveals an unexpected role exerted through recognition of the methylated CpGs of the ICRs that may have an essential role in maintaining the integrity of these regulatory regions and in the evolution of genomic imprinting in mammalian species.

## Materials and methods

### Cell lines and culture conditions

Mouse wild-type, *Zfp57*-/- and *Msh2*-/- E14 ESCs were cultured under standard feeder-free conditions on gelatinized tissue culture dishes and maintained in DMEM (Gibco, Thermo Fisher Scientific) supplemented with 100 uM 2-mercaptoethanol (Sigma), 1 mM sodium pyruvate, 2 mM l-glutamine, 1 × penicillin–streptomycin, 15% fetal calf serum (HyClone) and 10^3^ U/ml leukemia inhibitory factor (LIF, Millipore). The wild-type hybrid ESC line JB1, which is (JF1 × C57BL/6) F1, and the JB1-derived *Zfp57-/-* ESC line were described previously [7, 36]. Wild-type and *Zfp57*-/- JB1 ESCs were cultured under standard feeder-free conditions on gelatinized tissue culture dishes with ESGRO Complete™ Plus Serum-free Clonal Grade 1i Medium (Merck Millipore) in the presence of 3 μM Gsk3 inhibitor CHIR99021. For H_2_O_2_ exposure, 0.5 mM H_2_O_2_ was diluted in PBS immediately before adding it to the media and cells were collected 30 min later. Cells were cultured at 37 °C under an atmosphere of 5% CO_2_.

### Cloning and transfections

The cDNAs encoding the full-length *Zfp57* and *Msh2* genes were cloned into the expression vector containing the AviTag sequence, under the control of the elongation factor-1 alpha (*EF-1 alpha*) promoter. For plasmid transfection, cells were transfected with pEF1-BirA-V5_His and the pEF-AviTag-Zfp57/Msh2 plasmids using Lipofectamine LTX according to the manufacturer's protocol (Thermo Fisher Scientific). Stably BirA transfected cells were selected with 0.25 mg/ml G418 (Life Technologies) and maintained in 0.1 mg/ml G418. Mock-transfected BirA-expressing E14 ESCs were used as control for MS analysis and Bio-ChIP. The primers used for cloning are listed in the Additional file 1: Table S4.

### Protein immunoprecipitation analysis

Two 10 cm dishes of cells were pelleted and resuspended in NP40 buffer (10 mM Tris–HCl pH 7.4, 10 mM NaCl, 3 mM MgCl_2_ and 0.5% NP-40) on ice and incubated for 20 min at 4 °C. Isolated nuclei were lysed in SDS lysis buffer (50 mM Tris–HCl pH 8.0, 10 mM EDTA and 1% SDS) for 10 min at 4 °C. After incubation at 95 °C for 10 min, the nuclei lysate was sonicated on ice and centrifuged for 10 min at maximum speed. 1 mg of proteins (for KAP1, MSH6 and IgG IP) were pre-cleared with 30 μl protein A/G agarose beads (SantaCruz) for 2 h at 4 °C on a rotating wheel. Anti-KAP1 antibody (Abcam ab10483), anti-MSH6 (Santa Cruz sc-137015) or mouse IgG were added to the pre-cleared lysate and incubated overnight at 4 °C on a rotating wheel. Proteins were precipitated with 50 μl protein A/G agarose beads for 1 h at 4 °C with rotation. For biotin tagged-ZFP57 and -MSH2 immunoprecipitation, 1 mg of proteins was incubated with 100 μl of streptavidin beads overnight at 4 °C on a rotating wheel. The agarose and streptavidin beads were then washed five times with 500 μl RIPA buffer (10 mM Tris–HCl pH 8.0, 140 mM NaCl, 1 mM EDTA, 0.5 mM EGTA, 0.1% sodium deoxycholate, 1% Triton X-100 and 0.1% SDS, protease inhibitor (cOmplete, EDTA-free, Roche Life Science). Beads elution was followed by Mass Spectrometry or Western Blot analyses. High resolution nano-LC–tandem mass spectrometry and MS data processing were carried out as previously reported [37]. Proteins that were pulled down by streptavidin and identified by mass spectrometry in mock-transfected BirA-expressing E14 ESCs (Additional file 1: Table S1b) were excluded from further analysis. The list of ZFP57 interactors identified by LC–MS/MS was imported into the STRING database [18]. Cluster analysis was performed using the “kmean clustering” option with “number of clusters” parameter set to 4. The edges between clusters were set as “Dotted line”. To explore the processes in which those proteins are involved, we performed a GO:BP enrichment analysis using the function gost of gProfiler2 R package v 0.2.1 [38] with default options. We selected all the terms involved in either DNA or RNA processes with an adjusted *p*-values < 0.05. The selected terms were plotted using the GOplot package v 1.0.2 [39].

### Western blotting

Proteins were eluted from beads by incubating them at 95 °C for 10 min in Laemmli buffer and resolved by 8% acrylamide gel. Samples were transferred onto PVDF membranes (Biorad Transblot). After 1 h of blocking in 5% w/v milk/TBST at RT, membranes were incubated with the primary antibodies anti-ZFP57 (Abcam ab45341), anti-KAP1 (Abcam ab10483), anti-MSH6 antibody (BD Biosciences,610,918), anti- MSH2 (Calbiochem, NA27) and anti-Actin (Sigma-Aldrich, A2066) overnight at 4 °C. The membranes were washed 3 × with TBST and incubated with the secondary antibody. Signals were visualized using an ECL method.

### Chromatin immunoprecipitation (ChIP)

ChIP for the analysis of biotin tagged-MSH2, MSH6, H3K9me3 and H3K4me3 binding, was performed on formaldehyde cross-linked chromatin isolated from cells grown on 10 cm dishes to ∼80% confluency. Briefly, the cells were detached by adding 0.05% trypsin at 37 °C for 3 min. Formaldehyde was added to approximately 3 × 10^7^ cells resuspended in Phosphate Buffered Saline (PBS) at final concentration of 1% and the cells were incubated at room temperature for 10 min with shaking. The reaction was stopped by the addition of glycine to a final concentration of 0.125 M. Cells were washed twice in ice-cold PBS, centrifuged and resuspended in lysis buffer 1 (50 mM HEPES pH 8, 10 mM NaCl, 1 mM EDTA, 10% Glycerol, 0.5% NP-40 and 0.25% Triton X-100) for 10 min at 4 °C. Isolated nuclei were lysed in lysis buffer 2 (10 mMTris-HCl pH 8.0, 200 mM NaCl, 1 mM EDTA and 0.5 mM EGTA) for 10 min at 4 °C. The chromatin was sheared in a sonication buffer (10 mM Tris–HCl pH 8.0, 100 mM NaCl, 1 mM EDTA, 0.5 mM EGTA, 0.1% sodium deoxycholate and 0.5% N-lauroylsarcosine) to an average size of 100–400 bp using the S220 Focused-ultrasonicator (Covaris). Sonicated chromatin was diluted in sonication buffer. Chromatin for MSH6, H3K9me3 and H3K4me3 ChIP was pre-cleared with 30 μl protein A/G agarose beads (SantaCruz) for 4 h at 4 °C on a rotating wheel. Streptavidin beads (Dynabeads MyOne streptavidin T1, ref 65,601, Invitrogen) and anti-MSH6 (Santa Cruz sc-137015), anti-H3K9me3 (Abcam 8898), anti-H3K4me3 (Abcam ab8580) antibodies or rabbit/mouse IgG were added to the pre-cleared chromatin and incubated overnight at 4 °C on a rotating wheel. Chromatin for MSH6, H3K9me3 and H3K4me3 ChIP was precipitated with 30 μl protein A/G agarose beads for 4 h at 4 °C with rotation. The beads were then washed five times with 500 μl RIPA buffer (10 mM Tris–HCl pH 8.0, 140 mM NaCl, 1 mM EDTA, 0.5 mM EGTA, 0.1% sodium deoxycholate, 1% Triton X-100 and 0.1% SDS) and once with each of the following buffers: WASH buffer (50 mM HEPES, 0.5% sodium deoxycholate, 1% Triton X-100, 1 mM EDTA, 500 mM NaCl and 0.2% NaN3), LiCl buffer (0.25 M LiCl, 0.5% NP-40, 0.5% sodium deoxycholate, 1 mM EDTA and 10 mMTris pH 8) and TE buffer (10 mM Tris pH 8, 1 mM EDTA). The bound chromatin was eluted in 100 μl TE buffer. Cross-links were reversed by incubation at 1 h at 37 °C with 1 μl RNAse cocktail (Ambion) and O/N at 60 °C after addition of 2.5 μl 20% SDS and 2.5 μl 20 mg/ml proteinase K (Sigma). The DNA was extracted by using the QIAquick Gel Extraction Kit (Qiagen). The immunoprecipitated or 1% input DNAs were analysed by real-time PCR using SBYR Green PCR Master Mix (Bio-Rad) on a CFXCONNECT Thermal Cycler (Bio-Rad). Each reaction was performed in biological duplicate with each duplicate averaged over at least two technical replicates. To evaluate significance, we applied an unpaired Student’s T-test. Asterisks indicate statistical significant differences in enrichment of DNA sequences with adjusted p-value: *p < 0.05, **p < 0.01, ***p < 0.001. Primers are listed in Additional file 1: Table S4.

### Allele-specific ChIP

MSH2 binding as well as histone H3K9me3 and H3K4me3 enrichment on the methylated and non-methylated alleles of *Inpp5f* and *Gnas* loci was assessed after direct sequencing of the bisulfite-treated and PCR-amplified immunoprecipitated DNA. MSH6 binding on the JF1 and B6 alleles of *Plagl1*, and *Inpp5f* was determined by typing the immunoprecipitated DNA for the SNPs present between the two parental genomes. The amplification products were sequenced (Eurofins Genomics) and the ratio between allele-specific DNAs was determined from the electropherogram. All the primers are listed in the Additional file 1: Table S4.

### NGS analysis

For the RRBS library preparation, we used 100–200 ng of genomic DNA according to Illumina's instructions. Libraries were generated and sequenced at IGA Technology Services (Italy), by using the NuGEN Ovation RRBS Methyl-Seq Library System and paired-end 150 bp sequencing mode on NovaSeq6000 (Illumina, San Diego, CA). After adapter trimming and quality step using TrimGalore v. 0.6.6 [https://github.com/FelixKrueger/TrimGalore] with the RRBS mode (–rrbs), we aligned the reads against the *Mus musculus* genome (mm10) using Bismark v.0.23.0 with default parameters [40]. We removed the duplicate reads using UMI-tools v.1.1.1 with default parameters [41] and then used Bismark methylation extractor to obtain the methylation level of the CpGs covered.

For ChIP-seq analysis, two nanograms of DNA from immunoprecipitated and input chromatin were used for Illumina library preparation. Libraries were generated and sequenced at IGA Technology Services (Italy), by using the NuGen Ovation Ultralow Library System v2 Kit and 150 bp paired-end sequencing on the Illumina NovaSeq6000 platform (Illumina, San Diego, CA). After checking that there were no bad quality base calls and adapter contaminations in the raw data, we aligned the reads against the Mus musculus genome (mm10) using Bowtie2 short read aligner v2.3.5.1 with default parameters [42]. We removed duplicate reads using Picard MarkDuplicates v2.22.9 [http://broadinstitute.github.io/picard/] and the multiple mapping reads, and we used only uniquely mapped reads for the rest of the study.

The DNA binding profiles of ZFP57 and KAP1 in the E14 ESCs were obtained from the GSE77744 dataset. The coordinates were converted into the mm10 genome by using CrossMap Python script [43]. For visualization in UCSC, we used tracks normalized by reads per million (RPM) generated by the GenomeCoverageBed tools of the BEDtools suite v2.292 [44]. To define the enriched regions, we used MACS2 algorithm with PE and -broad parameters [45]. We performed the intersection of the peaks using Bedtools intersect function and evaluated its statistical significance using the function enrichPeakOverlap of the Bioconductor package ChIPseeker with nShuffle = 1000. The MSH2 peaks shared by the two replicates were 33% of the replicate 1 peaks and 62% of the replicate 2 peaks. The overlap (4444 peaks) was considered statistically significant. (*p* = 0.0001). We annotated the peaks using the ChIPseeker package [46] to the TxDb.Mmusculus.UCSC.mm10.knownGene database [47]. Using the plotAvgProf function with default parameters, we plotted the peaks frequency profile near the TSS. Moreover, for feature annotation, we used the plotAnnoPie function with annoDb = "org.Mm.eg.db" parameter. The statistical significance of the overlap between MSH2 peaks and promoters (−1000 + 0 of TSS) was calculated using the enrichPeakOverlap function and found to be highly significant (*p* = 0.0001). Concerning annotation of the CpG islands and Rmsk regions, we intersected the peaks with the coordinates of those regions downloaded from UCSC. We plotted the heatmap of scores associated with genomic regions using the computeMatrix and plotHeatmap function of the deepTools v3.4.3 [48]. The raw and processed files are deposited in GEO under the accession number GSE205043. Publicly available datasets of KAP1 and ZFP57 ChIPseq were sourced from GEO database: GSE77444.

## Supplementary Information

**Additional file 1:** **Table S1.** List of high-confidence proteins pulled down with ZFP57-AviTag in E14 ESCs and identified by nano-LC–MS/MS. **Table S1b.** List of shared proteins pulled down with streptavidin in E14 BirA (controls) and E14 BirA+ZFP57_Avitag (samples). **Table S2a**. MSH2 peaks identified by Bio-ChIP-seq in E14 ESCs. Replicate 1. **Table S2b.** MSH2 peaks identified by Bio-ChIP-seq in E14 ESCs. Replicate 2. **Table S3.** Coordinates of the imprinted gDMRs in mouse ESCs*. **Table S4.** Primer sequences.

**Additional file 2:** **Fig. S1.** STRING network model of ZFP57-interacting proteins. 41 of the 60 candidate ZFP57-interacting proteins were mapped on an interconnected network constructed by STRING analysis. The model revealed key sub-network clusters connected to ZFP57 and KAP1(TIF1B). Colours represent different subnetworks based on K-means clustering. Edge thickness is representative of the confidence in interaction based on database mining, experimental evidence and text mining. **Fig. S2.** Heat-map showing that the great majority of the MSH2 Bio-ChIP-seq peaks overlapping with promoters are centered on Transcription Start Sites (TSS). **Fig. S3**. Screenshots from the UCSC Genome Browser showing the ChIP-seq signals detected for biotin-tagged MSH2 in BirA-expressing E14 ESCs along eight cell growth-controlling genes with promoters overlapping CpGI. DNA methylation and binding profiles of MSH2 (2 replicates), ZFP57 and KAP1 are reported as in Figure 3a. **Fig. S4**. (related to Figure 2f) Heatmaps showing the read enrichment of MSH2, MSH6, ZFP57 and KAP1 in the genomic regions overlapping (+/- 1.5 kbp) the KAP1 ChIP-seq peaks sorted on the basis of their overlap with various genomic elements. **Fig. S5.** (related to Figure 2f) Heatmaps showing the read enrichment of MSH2, MSH6, ZFP57 and KAP1 in the genomic regions overlapping (+/- 1.5 kbp) the KAP1 ChIP-seq peaks sorted on the basis of their overlap with ZFP57 peaks. **Fig. S6**. Screenshots from the UCSC Genome Browser showing the ChIP-seq signals detected for the Biotin-tagged MSH2 in BirA-expressing E14 ESCs along five noICR regions bound by ZFP57 [7]. DNA methylation and binding profiles of MSH2 ( 2 replicates), ZFP57 and KAP1 are reported as in Figure 3a.

## Data Availability

Raw data supporting the findings of this study have been deposited under accession code GSE205043 in the Gene Expression Omnibus repository.
