# Supplementary material for: The mismatch-repair proteins MSH2 and MSH6 interact with the imprinting control regions through the ZFP57-KAP1 complex
Source: Epigenetics Chromatin. 2022 Aug 2;15:27. doi: 10.1186/s13072-022-00462-7 (PMC9344765; doi:10.1186/s13072-022-00462-7)
Supplement: Supplementary file 2 — Additional file 2: Fig. S1. STRING network model of ZFP57-interacting proteins. 41 of the 60 candidate ZFP57-interacting proteins were mapped on an interconnected network constructed by STRING analysis. The model revealed key sub-network clusters connected to ZFP57 and KAP1(TIF1B). Colours represent different subnetworks based on K-means clustering. Edge thickness is representative of the confidence in interaction based on database mining, experimental evidence and text mining. Fig. S2. Heat-map showing that the great majority of the MSH2 Bio-ChIP-seq peaks overlapping with promoters are centered on Transcription Start Sites (TSS). Fig. S3. Screenshots from the UCSC Genome Browser showing the ChIP-seq signals detected for biotin-tagged MSH2 in BirA-expressing E14 ESCs along eight cell growth-controlling genes with promoters overlapping CpGI. DNA methylation and binding profiles of MSH2 (2 replicates), ZFP57 and KAP1 are reported as in Figure 3a. Fig. S4. (related to Figure 2f) Heatmaps showing the read enrichment of MSH2, MSH6, ZFP57 and KAP1 in the genomic regions overlapping (+/- 1.5 kbp) the KAP1 ChIP-seq peaks sorted on the basis of their overlap with various genomic elements. Fig. S5. (related to Figure 2f) Heatmaps showing the read enrichment of MSH2, MSH6, ZFP57 and KAP1 in the genomic regions overlapping (+/- 1.5 kbp) the KAP1 ChIP-seq peaks sorted on the basis of their overlap with ZFP57 peaks. Fig. S6. Screenshots from the UCSC Genome Browser showing the ChIP-seq signals detected for the Biotin-tagged MSH2 in BirA-expressing E14 ESCs along five noICR regions bound by ZFP57 [7]. DNA methylation and binding profiles of MSH2 ( 2 replicates), ZFP57 and KAP1 are reported as in Figure 3a. [file 13072_2022_462_MOESM2_ESM.pdf]

Supplementary Figures.

Fig. S1 STRING network model of ZFP57-interacting proteins. 41 of the 60 candidate ZFP57-interacting proteins were mapped on an interconnected network constructed by STRING analysis. The model revealed key sub-network clusters connected to ZFP57 and KAP1(TIF1B). Colours represent different subnetworks based on K-means clustering. Edge thickness is representative of the confidence in interaction based on database mining, experimental evidence and text mining.

Fig. S2 Heat-map showing that the great majority of the MSH2 Bio-ChIP-seq peaks overlapping with promoters are centered on Transcription Start Sites (TSS).

Fig. S3 Screenshots from the UCSC Genome Browser showing the ChIP-seq signals detected for biotin-tagged MSH2 in BirA-expressing E14 ESCs along eight cell growth-controlling genes with promoters overlapping CpGI. DNA methylation and binding profiles of MSH2 (2 replicates), ZFP57 and KAP1 are reported as in Figure 3a.

Fig. S4 (related to Figure 2f) Heatmaps showing the read enrichment of MSH2, MSH6, ZFP57 and KAP1 in the genomic regions overlapping (+/- 1.5 kbp) the KAP1 ChIP-seq peaks sorted on the basis of their overlap with various genomic elements.

Fig. S5 (related to Figure 2f) Heatmaps showing the read enrichment of MSH2, MSH6, ZFP57 and KAP1 in the genomic regions overlapping (+/- 1.5 kbp) the KAP1 ChIP-seq peaks sorted on the basis of their overlap with ZFP57 peaks.

Fig. S6 Screenshots from the UCSC Genome Browser showing the ChIP-seq signals detected for the Biotin-tagged MSH2 in BirA-expressing E14 ESCs along five non-ICR regions bound by ZFP57 [7]. DNA methylation and binding profiles of MSH2 (2 replicates), ZFP57 and KAP1 are reported as in Figure 3a.

Fig. S1

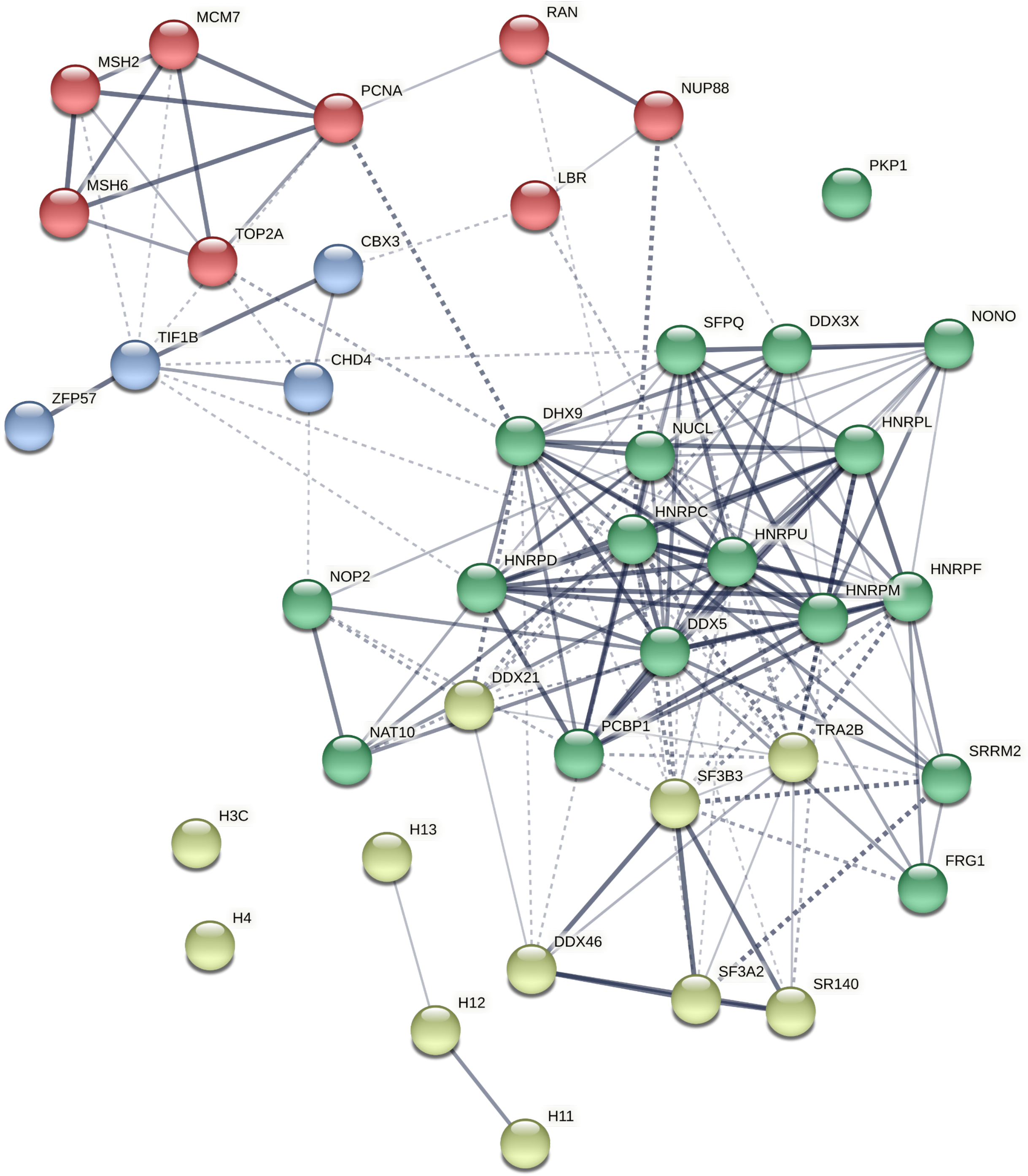
MCM7
RAN
MSH2
PCNA
NUP88
PKP1
MSH6
LBR
TOP2A
CBX3
TIF1B
CHD4
ZFP57
SFPQ
DDX3X
NONO
DHX9
NUCL
HNRPL
HNRPC
HNRPU
HNRPD
HNRPF
NOP2
HNRPM
DDX5
DDX21
NAT10
PCBP1
TRA2B
SRRM2
SF3B3
H3C
H13
FRG1
H4
DDX46
SF3A2
SR140
H12
H11

Fig. S2

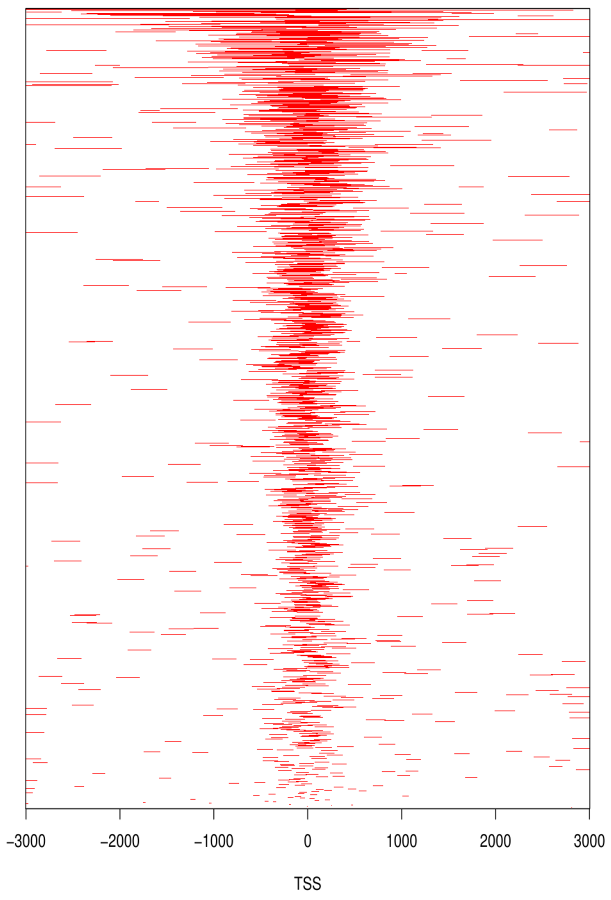

Fig. S3

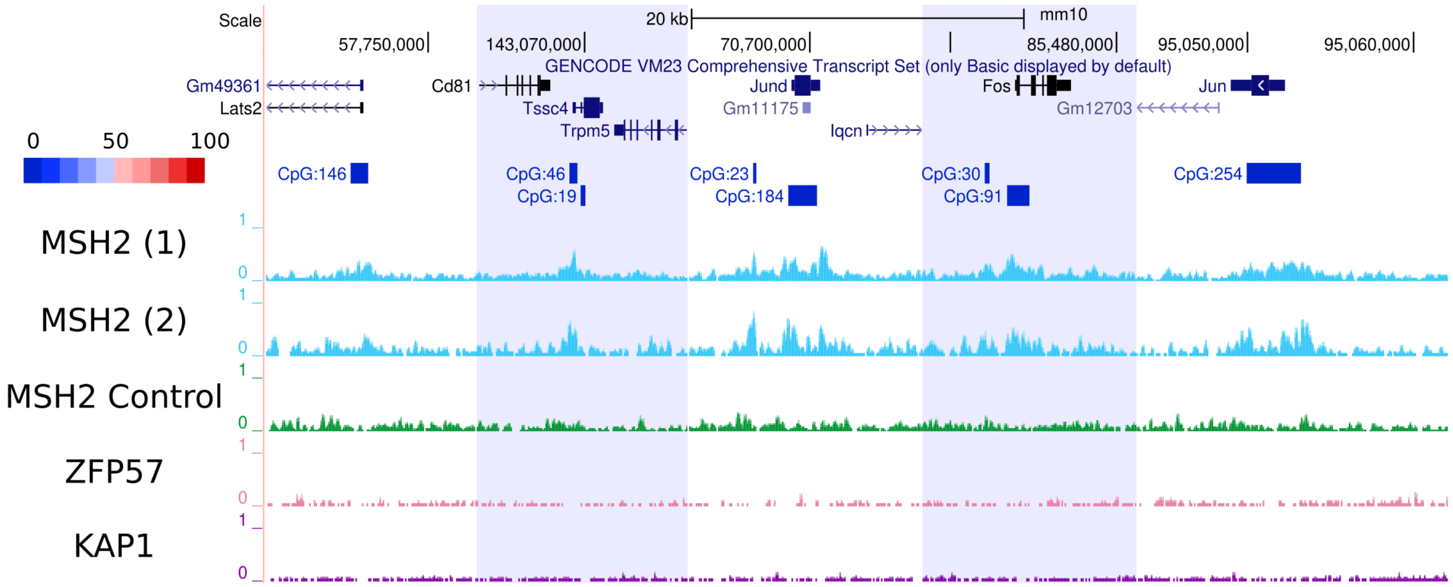

Scale
20 kb
mm10
57,750,000
143,070,000
70,700,000
85,480,000
95,050,000
95,060,000
GENCODE VM23 Comprehensive Transcript Set (only Basic displayed by default)
Gm49361
Lats2
Cd81
Tssc4
Trpm5
Jund
Gm11175
Iqcn
Fos
Gm12703
Jun
0
50
100
CpG:146
CpG:46
CpG:19
CpG:23
CpG:184
CpG:30
CpG:91
CpG:254
MSH2 (1)
MSH2 (2)
MSH2 Control
ZFP57
KAP1

Fig. S4

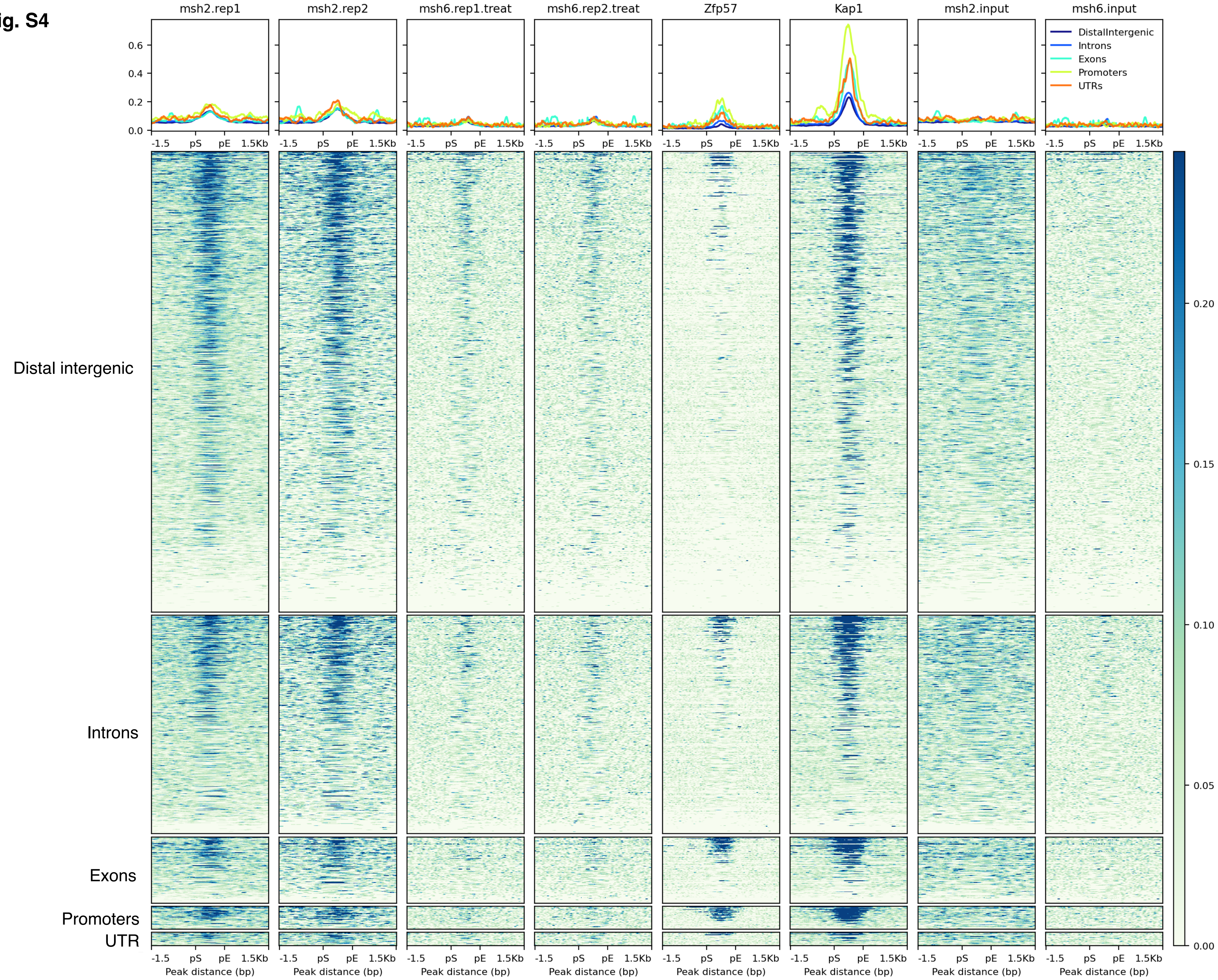

msh2.rep1
msh2.rep2
msh6.rep1.treat
msh6.rep2.treat
Zfp57
Kap1
msh2.input
msh6.input
DistalIntergenic
Introns
Exons
Promoters
UTRs
0.0
0.2
0.4
0.6
-1.5
pS
pE
1.5Kb
Distal intergenic
Introns
Exons
Promoters
UTR
Peak distance (bp)
0.00
0.05
0.10
0.15
0.20

Fig. S5

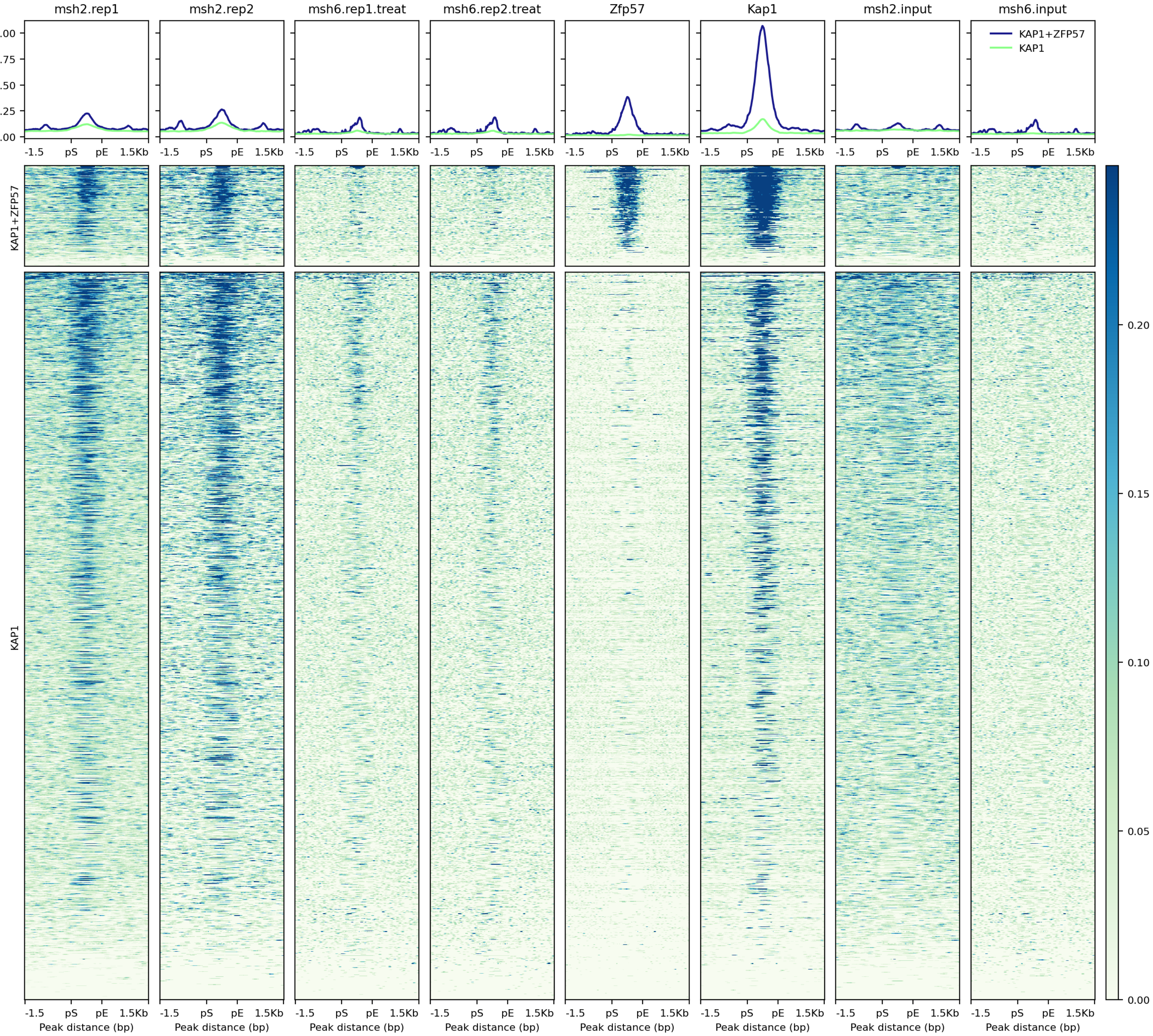

msh2.rep1
msh2.rep2
msh6.rep1.treat
msh6.rep2.treat
Zfp57
Kap1
msh2.input
msh6.input
KAP1+ZFP57
KAP1
1.00
0.75
0.50
0.25
0.00
-1.5
pS
pE
1.5Kb
Peak distance (bp)
0.20
0.15
0.10
0.05
0.00

Fig. S6

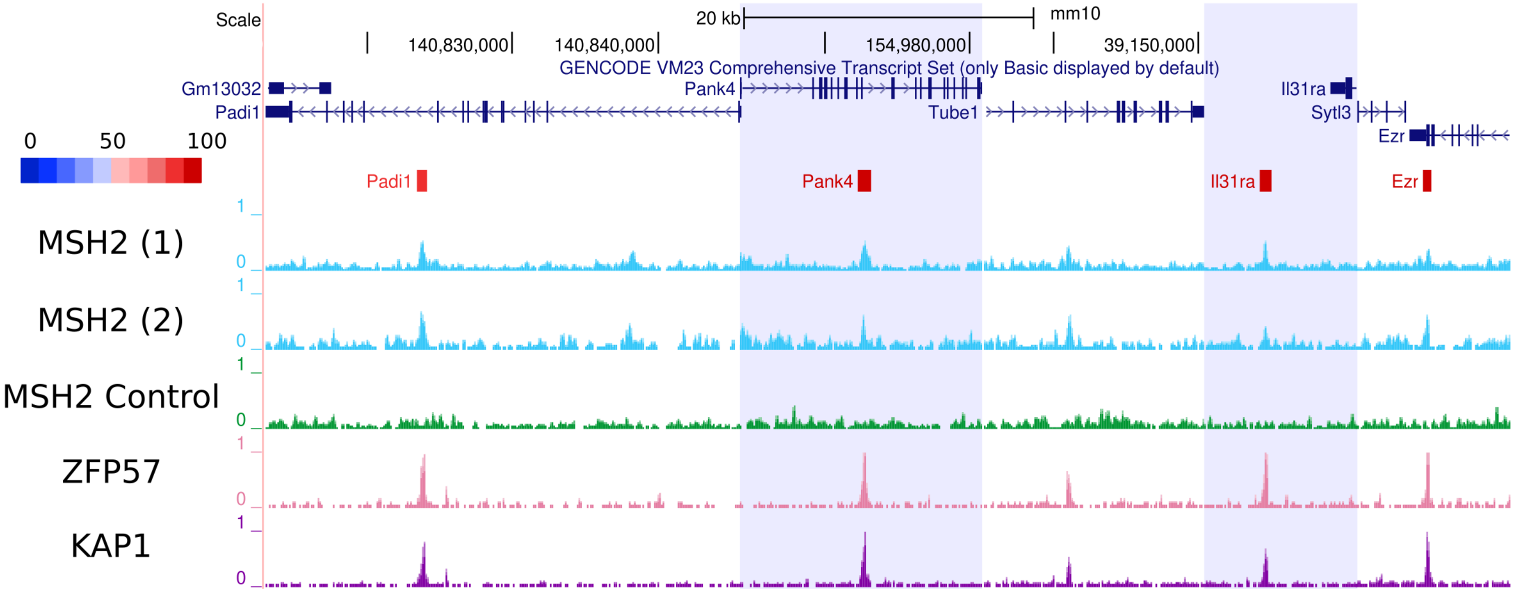

Scale
20 kb
mm10
140,830,000
140,840,000
154,980,000
39,150,000
GENCODE VM23 Comprehensive Transcript Set (only Basic displayed by default)
Gm13032
Padi1
Pank4
Tube1
Il31ra
Sytl3
Ezr
0
50
100
Padi1
Pank4
Il31ra
Ezr
MSH2 (1)
MSH2 (2)
MSH2 Control
ZFP57
KAP1
